# Localized states and quantum effect of photo-generated carriers in photovoltaic system

**DOI:** 10.1038/s41598-017-07663-1

**Published:** 2017-08-03

**Authors:** Wei-Qi Huang, Shi-Rong Liu, Zhong-Mei Huang, Xue-Ke Wu, Chao-Jian Qin

**Affiliations:** 1 Institute of Nanophotonic Physics, Guizhou University, Guiyang, 550025 China; 2 State key laboratory of Surface Physics, Key Laboratory of Micro and Nano Photonic Structures (Ministry of Education) and Department of Physics, Fudan University, Shanghai, 200433 China; 3 State Key Laboratory of Ore Deposit Geochemistry Institute of Geochemistry, Chinese Academy of Science Institute of Geochemistry, Guiyang, 550003 China

## Abstract

We have fabricated the multiple nanolayers impuritied on silicon pillars for Si solar cells to pick up photons in ultraviolet and infrared region of solar spectra, in which the localized states originated from nanosilicon doped with oxygen are built to avoid Auger recombination, and some interesting quantum phenomena in the localized states have been observed. The quantum effect of photo-generated carriers has been observed in I-V curve measurement on the photovoltaic sample prepared in oxygen by using nanosecond pulsed laser. More interesting, the twin states of quantum vibration are measured in the localized states originated from the impuritied nanosilicon, which provides a stable reservoir for electrons in the photovaltaic system. It should be noted that the amplitude change of the quantum vibration occurs under magnetic field with 0.33T on the sample prepared in oxygen, owing to the electron spin in the localized states. The photoluminescence (PL) spectra measured from 300 nm to 1700 nm exhibit the localized states in various regions in the photovoltaic system, in which the electrons can stand in the localized states with longer lifetime to be uneasy into Auger recombination.

## Introduction

Surface-induced effects on micro-nanopattern and quantum confinement effect in Si nanostructures offer interesting features that could be used to boost the efficiency of photovoltaic energy conversion and to overcome some of the restraints that lead to the Shockley–Queisser limit^1–5^. Micro-nanostructuring has been suggested as a promising method to find a new path to get an effective absorber for solar cells with higher efficiency in a photovoltaic system^6–21^. Recently, significant effort has been focused on enhancing the light absorption by nanoscale light trapping using nanowires, nanocones, nanodomes and nanoholes^22–31^. Despite the exciting success in light trapping, the power conversion efficiency of nano-structured Si solar cells, however, remain lower efficiency for the thick devices and the thin devices^32, 33^. The Si solar cells with nanostructures are not efficient because of severe Auger recombination. In addition it is also needed for Si solar cells with higher efficiency to pick up photons in ultraviolet and infrared region of solar spectra.

Here, we have found the new methods in which the localized electronic states with longer lifetime due to the Heisenberg principle related to Δt ~ h/ΔE^31^ are built from the impurities on the nanostructures for avoiding severe Auger recombination, which involve the electronic states due to impurities built on the smaller nanostructures doped with oxygen and the electronic states owing to impurities built on the defects doped with oxygen for improving photovoltaic conversion in ultraviolet and infrared regions.

In the article, the quantum effect of photo-generated carriers in the localized states is observed in I-V curve measurement on the photovoltaic sample prepared in oxygen, which provides a stable reservoir for electrons. It is very interesting that the quantum vibration on the quantum platform of I-V curve is discovered on the Si quantum dots (QDs) doped with oxygen, and the quantum twin-state occurs in the localized states under irradiation of laser at 633 nm. The amplitude change of the quantum vibration occurring in the magnetic field with 0.33T should be related to the electron spin in the localized states. The quantum twin-state in the localized states originated from the QDs impuritied should have an application in quantum entangle and code.

The photoluminescence (PL) spectra measured from 300 nm to 1700 nm exhibit the electronic states in various regions, especially the localized states for avoiding Auger recombination on the photovoltaic system, in which the electrons can stand in the localized states with longer lifetime originated from the impurities on the nanosilicon or on the defects of silicon to be uneasy into Auger recombination.

**Figure 1:**
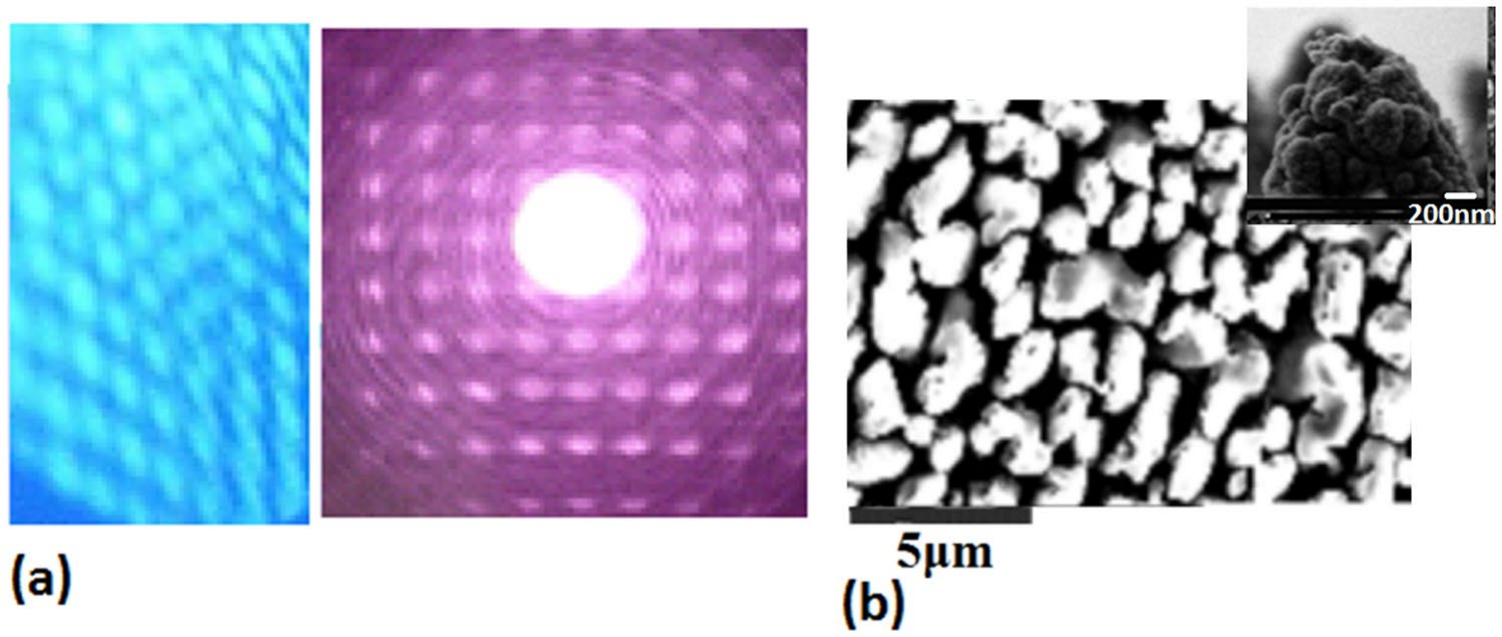
(**a**) In the PLE process, the plasmonic lattice structure induced by ns-laser as shown in the left optical image, and the diffraction pattern on the plasmonic lattice structure (similar with Wigner crystal structure) as exhibited in the right optical image; (**b**) The SEM image showing the pillars structure prepared by using ns-laser, where the inset exhibits the detail shape on the single tip of pillars.

We use the pulsed laser etching (PLE) device with a ns laser to fabricate the pillars structure, in which the spot diameter of laser beam is about 1 μm focused on the silicon wafers of P-type substrate with 10 Ωcm in vacuum (sample I) or in oxygen environment with 80 Pa (sample II). It can be assumed that the pure nanosilicon occurs in the sample I, and the sample II involves the nanosilicon doped with oxygen. It is interesting that the plasma lattice pattern occurs in the process of etching the pillars structure as shown in Fig. 1(a), in which the left optical image exhibits the plasmonic lattice structure induced by ns-laser and the right optical image shows the diffraction pattern on the plasmonic lattice structure (similar with Wigner crystal structure). After etching, the suitable annealing process on the pillars structure is important for its crystallizing to form the nanostructures and defects. The SEM image in Fig. 1(b) shows the pillars structure prepared by using ns-laser, where the inset exhibits the cross sectional shape in nanoscale on the single tip of pillars. The refractive index is about 1.88 in visible range on the SiO_2_ surface of the pillars and the experimental result obeys the K-K relations^34^.

**Figure 2:**
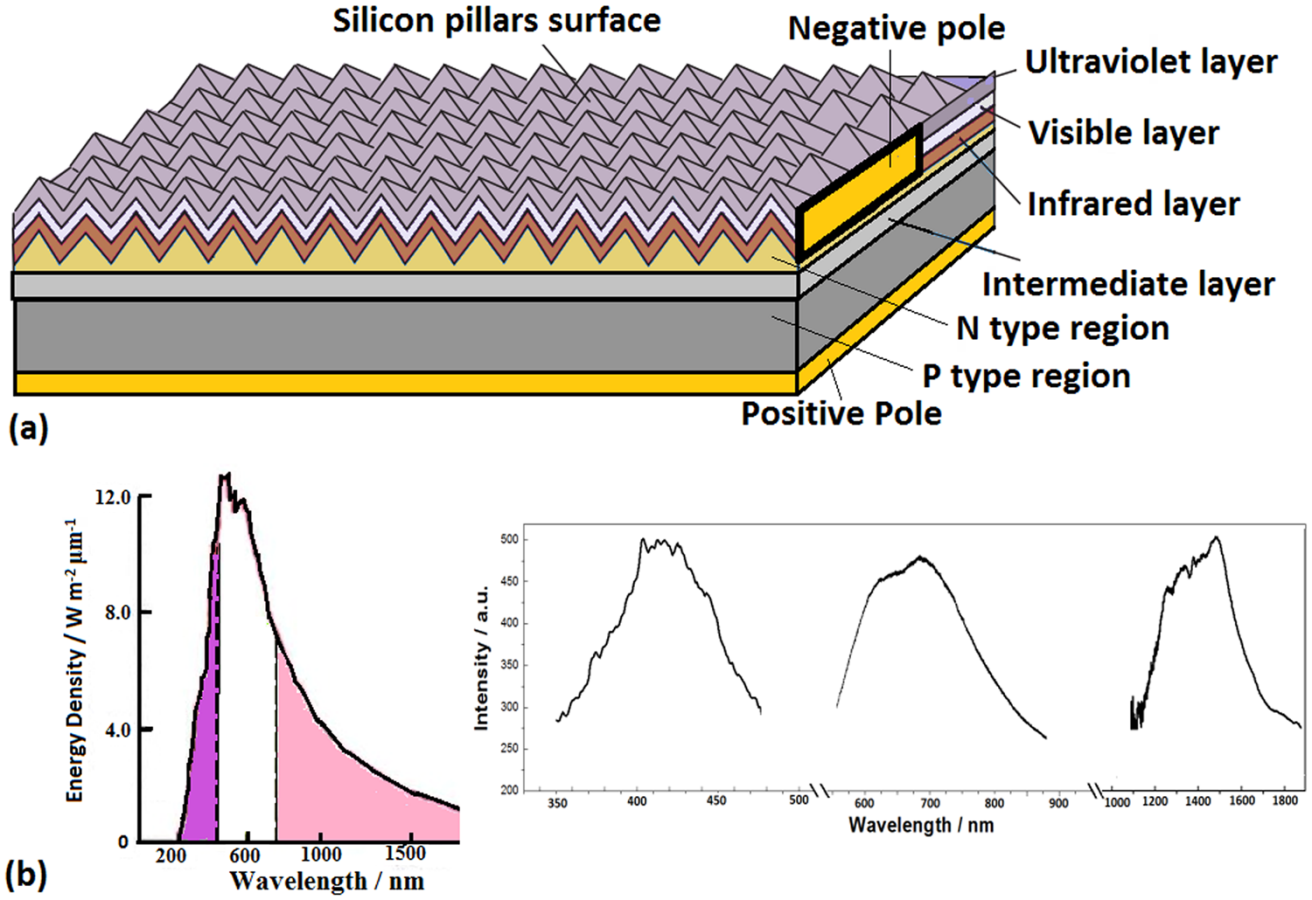
(**a**) The three layers structure of photovoltaic system involving ultraviolet layer, visible layer and infrared layer built under the silicon pillars surface; (**b**) The solar spectrum and the spectra related to the electronic states in the inset showing the ultraviolet spectrum in the ultraviolet layer, the visible spectrum in the visible layer and the infrared spectrum in the infrared layer.

As shown in Fig. 2(a), the three layers involving ultraviolet layer, visible layer and infrared layer are built under pillars surface. The localized states from the smaller nanosilicon doped with oxygen in the ultraviolet layer can transfer the ultraviolet photons into pairs of electron and hole. The localized states from the bigger nanosilicon doped with oxygen in the visible layer can hold carriers by absorbing visible photons. And the localized electronic states from defects doped with oxygen in the infrared layer can transfer the infrared photons into carriers. The combinational transformation of the energy states in the three layers conformation can almost cover the solar spectra, as shown in Fig. 2(b), in which the inset shows the ultraviolet spectrum in the ultraviolet layer, the visible spectrum in the visible layer and the infrared spectrum in the infrared layer.

**Figure 3:**
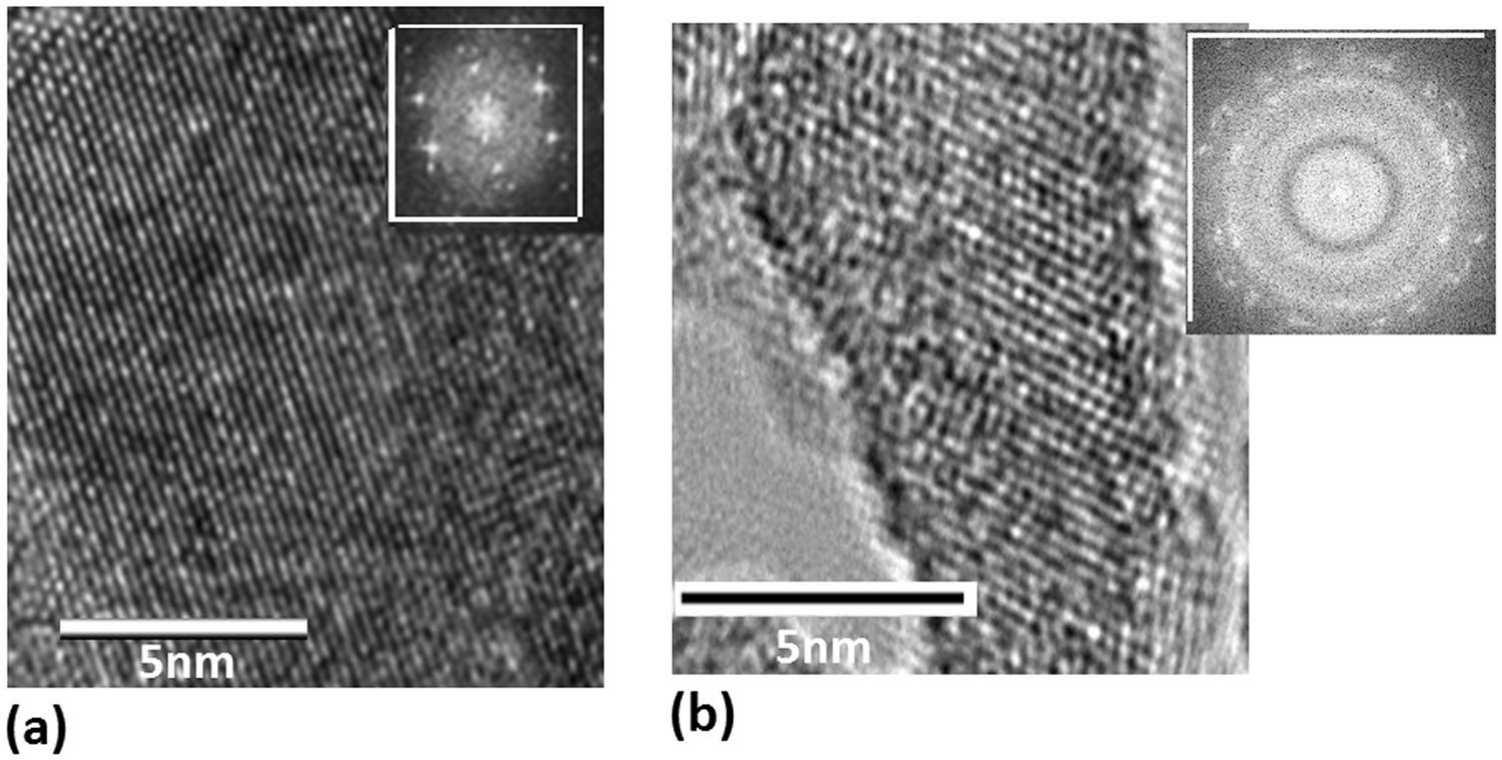
(**a**) TEM image of Si nanolayer prepared in vacuum, where the inset shows FFT pattern of silicon crystal after annealing for 30 min; (**b**) TEM image of Si nanolayer prepared in oxygen of 80 Pa, in which the FFT pattern of the inset exhibits the broken symmetry of silicon crystal doped with oxygen after annealing for 30 min.

For confirmation of oxygen doping in nanosilicon, the comparison between the sample I prepared in vacuum and the sample II prepared in oxygen environment with 80 Pa has been taken in the TEM images. In Fig. 3(a), the TEM image of Si nanolayer prepared in vacuum exhibits the pure silicon occurs in the sample I after annealing for 30 min, and the inset shows FFT pattern of silicon crystal. The TEM image of Si nanolayer doped with oxygen after annealing for 30 min shows the impurities structure of the sample II prepared in oxygen environment with 80 Pa, where the stronger condensed structure of Si-O bonds occurs on the surface of Si nanolayer, as shown in Fig. 3(b), in which the FFT pattern of the inset exhibits the broken symmetry of silicon crystal doped with oxygen.

## Localized states effect on nanosilicon doped with oxygen

**Figure 4:**
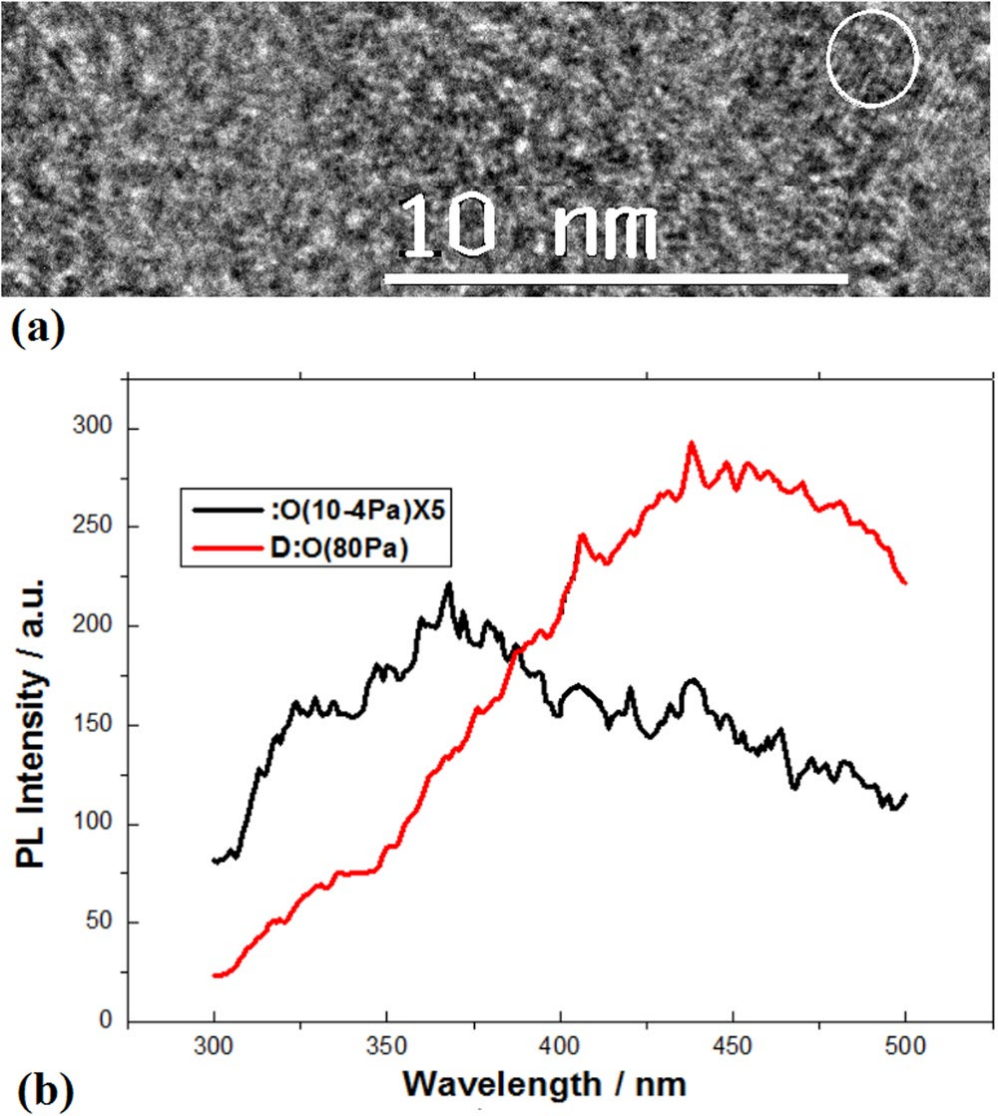
(**a**) TEM image showing the smaller nanosilicon doped with oxygen in the ultraviolet layer; (**b**) PL spectra on the sample I (black curve) prepared in vacuum and on the smple II (red curve) prepared in oxygen of 80 Pa, in which PL peak near 450 nm is related to the localized electronic states owing to the smaller Si QDs doped with oxygen.

The PL spectrum from 350 nm to 500 nm measured in the ultraviolet layer exhibits the localized states from the smaller nanosilicon doped with oxygen. Here, the localized states originated from impurities on nanosilicon effectively avoid the Auger recombination on the photovoltaic system. In Fig. 4(a), the TEM image shows the smaller QDs of silicon doped with oxygen in the ultraviolet layer. And the PL peak near 450 nm related to the localized states is shown in Fig. 4(b), in which it is interesting to make a comparison for PL spectra on the sample I (black curve) prepared in vacuum and on the sample II (red curve) prepared in oxygen of 80 Pa. Here, the enhanced broader peak (red curve) near 450 nm is related to the localized states emission on the smaller QDs doped with oxygen.

**Figure 5:**
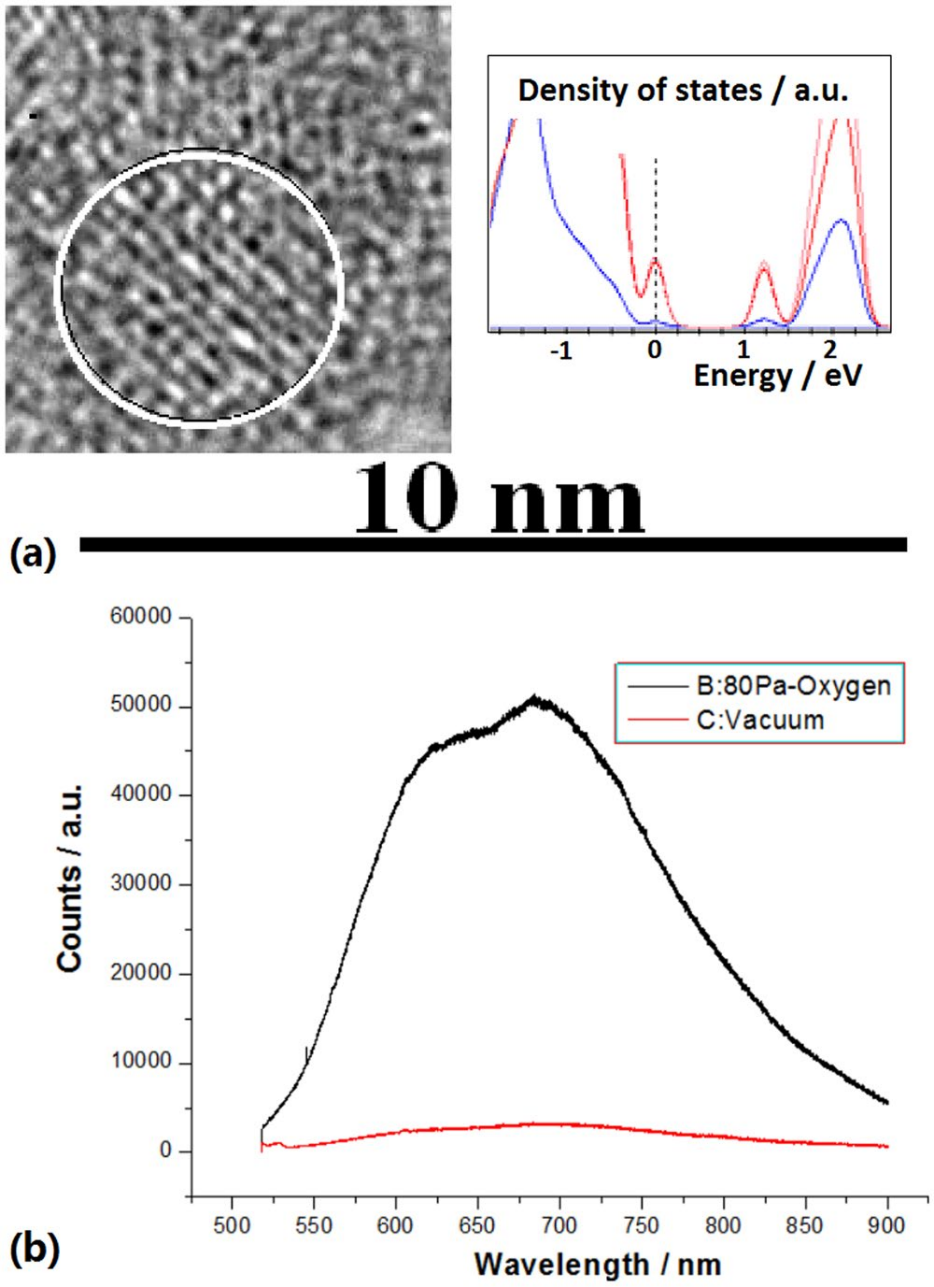
(**a**) The TEM image showing the bigger Si QDs doped with oxygen of 80 Pa, and the inset showing the density distribution of states with the localized states originated from impurities on nanocrystals in the simulating calculation; (**b**) The comparison for the PL spectra from 550 nm to 850 nm measured in the visible layer, in which the red curve is related to the spectrum on the sample I prepared in vacuum and the black curve is related to the spectrum on the sample II prepared in oxygen owing to the localized electronic states from the bigger nanocrystals doped with oxygen.

The TEM image in Fig. 5(a) shows the bigger nanosilicon doped with oxygen of 80 Pa, where the Si-O bonds are obvious on the Si QDs, and the inset shows the density distribution of states with the localized states originated from impurities on nanosilicon in the simulating calculation, which effectively avoid the Auger recombination on the photovoltaic system due to the carriers have a longer lifetime in the localized states. In Fig. 5(b), the comparison for the PL spectra from 550 nm to 850 nm measured in the visible layer exhibits that the localized states from the bigger QDs doped with oxygen occur on the sample II prepared in oxygen related to the black curve in the spectra.

## Impurities states effect on silicon defects

**Figure 6:**
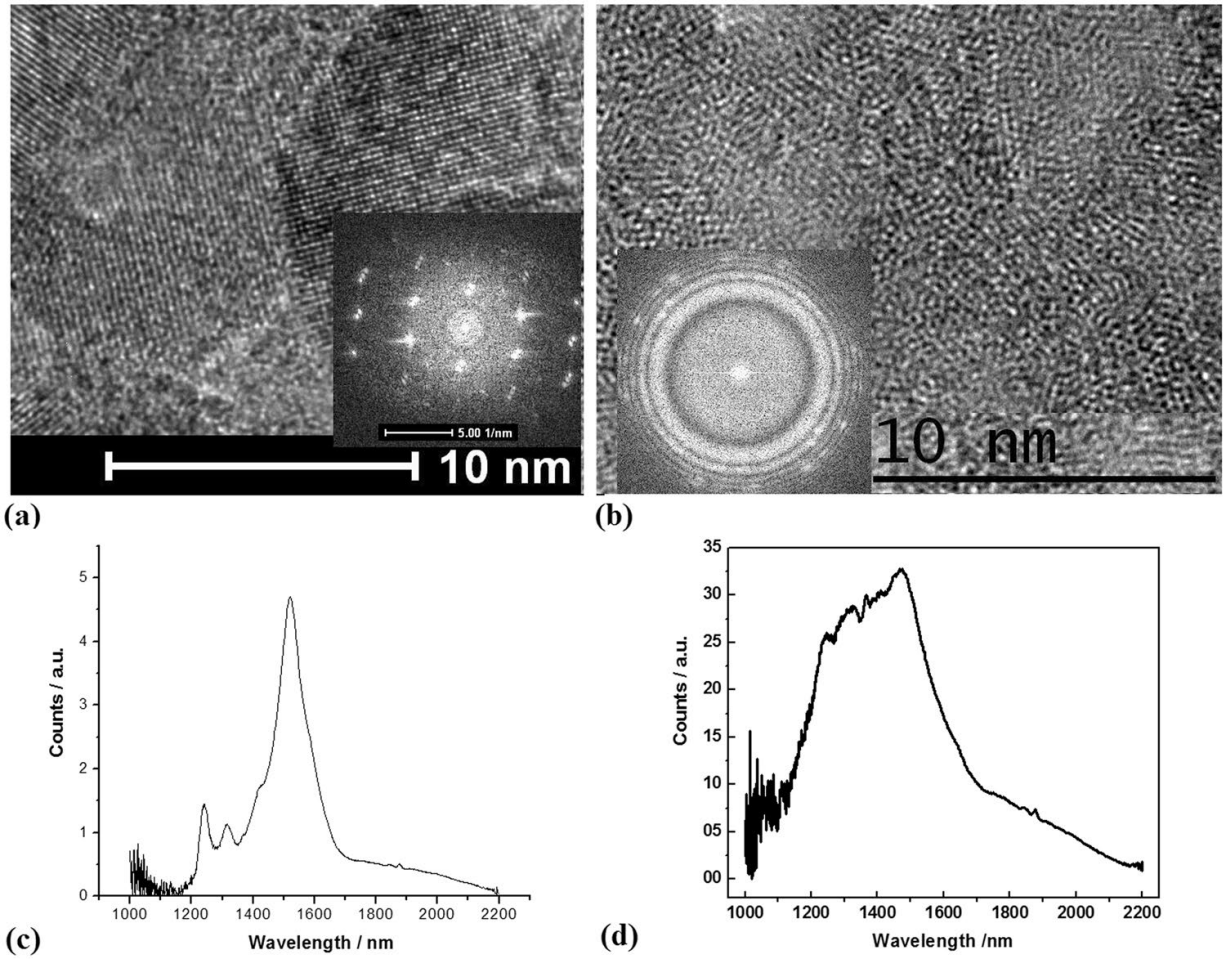
(**a**) The TEM image of the crystal structures of the pillars sample I prepared in vacuum and their FFT pattern shown in the inset, which exhibits the various defects; (**b**) The TEM image of the crystal structures of the pillars sample II prepared in oxygen of 80 Pa and their FFT pattern shown in the inset, which exhibits the defects doped with oxygen; (**c**) The PL spectrum on the sample I indicating the defects states from D1 to D4; (**d**) The PL spectrum on the sample II prepared in oxygen showing the broader impurities states near the defects states.

It is interesting to make a comparison of electron states between the pillars samples prepared in vacuum and in oxygen of 80 Pa, whose PL spectra on the two kinds of samples are exhibited in Fig. 6. In Fig. 6(a), the TEM image shows the crystal structures of the pillars sample I prepared in vacuum, and their FFT pattern is shown in the inset, which exhibits the various defects. In contrast to the sample I, the TEM image of Fig. 6(b) shows the crystal structures of the pillars sample II prepared in oxygen of 80 Pa, their FFT pattern is shown in the inset, which exhibits the defects doped with oxygen. In Fig. 6(c), the PL spectrum on the pillars sample I indicate the defects states from D1 to D4 respectively related to the peaks near 1250 nm, 1310 nm, 1400 nm and 1540 nm. And in Fig. 6(d), the PL spectrum on the pillars sample II prepared in oxygen exposures the broader impurities states near the defects states.

## Quantum effect of photo-generated carriers

**Figure 7:**
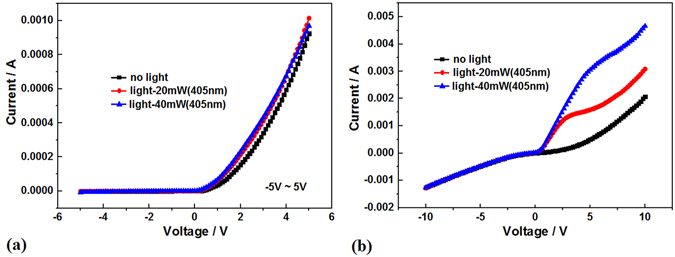
(**a**) The I-V curves in dark (black curve) and in light field (red and blue curve) of laser at 405 nm on the pillars sample I prepared in vacuum; (**b**) The I-V curves in dark (black curve) and in light field (red and blue curve) of laser at 405 nm on the pillars sample II prepared in oxygen, in which the quantum effect of photo-generated carriers obviously occurs.

**Figure 8:**
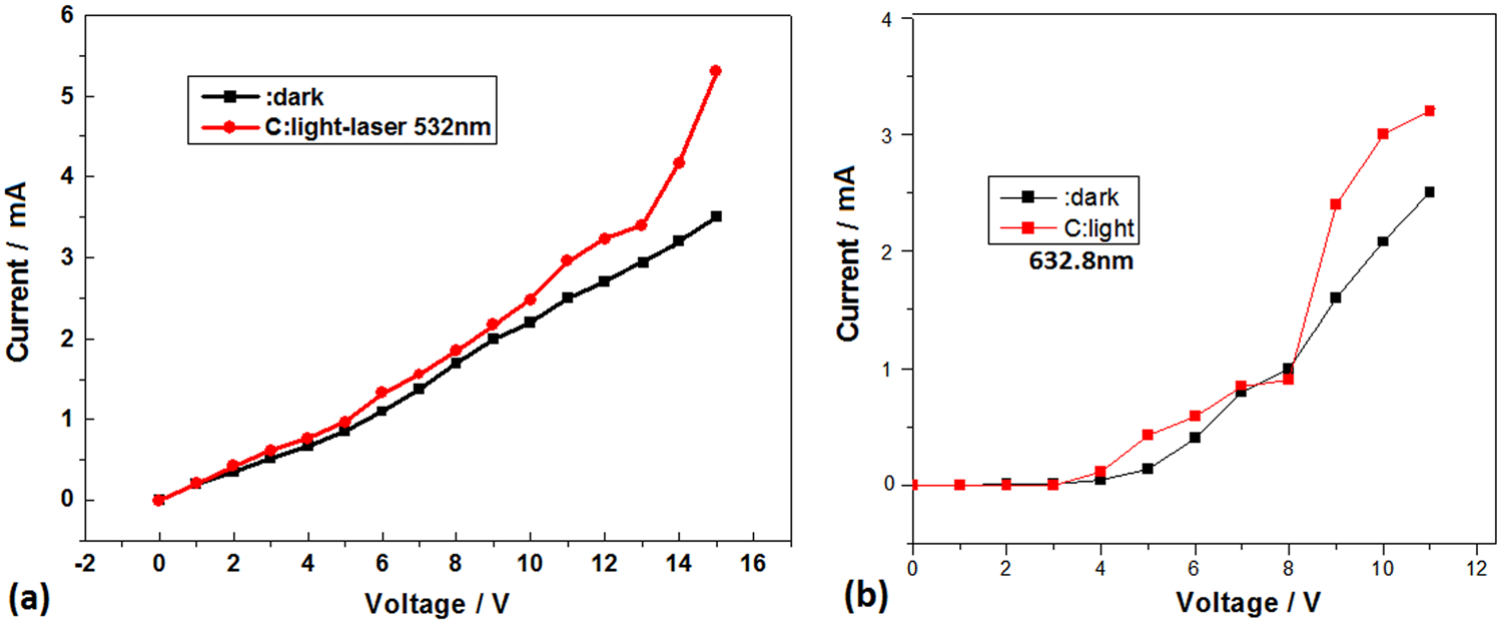
(**a**) The I-V curves in dark (black curve) and in light field (red curve) of laser at 532 nm on the pillars sample II prepared in oxygen; (**b**) The I-V curves in dark (black curve) and in light field (red curve) of laser at 633 nm on the pillars sample II prepared in oxygen.

In the photovoltaic measurement, much more photo-generated carriers under irradiation obviously occur on the sample II prepared in oxygen, as shown in Fig. 7. More interesting for making a comparison between Fig. 7(a) and (b), in the localized states, much more photo-generated carriers produced by laser at 405 nm obviously appear in the photo-generated current curve just on the sample II prepared in oxygen of 80 Pa; but the photo-generated current is very weaker on the sample I prepared in vacuum. The quantum effect of photo-generated carriers obviously occurs as shown in Fig. 7(b), where the two quantum steps in the I-V curve occur with laser irradiation power at 20 mW and 40 mW, in which the quantum platform is built in the localized states owing to exhausted photo-generated carriers in nanolayer with certain irradiation photons on the sample II. The changing of quantum steps with irradiation photons in the I-V curves could be applied for photo-amplifier. In the same way, the quantum effect of photo-generated carriers also appears in the I-V curves on the sample II under irradiation of laser at 532 nm and 632.8 nm respectively, as shown in Fig. 8(a) and (b). The quantum effect of photo-generated carriers involves the two processes, at first the photo-generated carriers jump up from the localized states and tunneling out to form the quantum step in which the voltage threshold decreases obviously, then the exhaustion of photo-generated carriers form the quantum platform.

In the experiments, the external quantum efficiency (EQE) was measured directly under laser radiation at 633 nm on the sample II and the sample I. The EQE on the sample II is about 70% which is ten times higher than the EQE on the sample I. The better inversion of photo-voltage on the sample II prepared in oxygen environment should be originated from the reservoir of carriers avoiding Auger recombination in the localized states due to the nanosilicon doped with oxygen.

It is very interesting that the localized states originated from impurities on nanosilicon is really the trap states with longer lifetime localizing under the opened conduction band of nanocrystals owing to the Heisenberg principle related to Δt ~ h/ΔE, in which the longer Δt can be obtained with the narrower ΔE in the localized states. The photo-generated electrons in the localized states flow out to form current, which has the quantum tunneling characteristic in the nanolayer doped with oxygen. The carriers density in the localized states can be described by the formula: N = Σ_i_ (4п mK_B_T/h) ln{1 + exp[(E_f_ − E_i_)/(K_B_T)]}ψ_i_ψ_i_^*^, where E_f_ is the Fermi energy level, E_i_ and ψ_i_ is energy value and wave function of the carriers in the localized state, respectively^35^.

**Figure 9:**
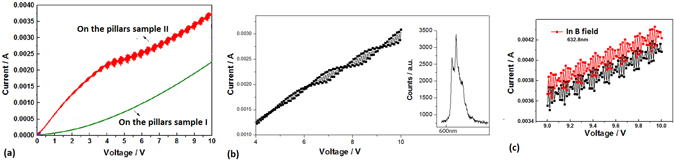
(**a**) The I-V curves in light field of laser at 405 nm on the pillars sample II prepared in oxygen (red curve) and on the Si wafer (green curve), in which the quantum vibration and the quantum platform in the I-V curve occur on the pillars structure doped with oxygen; (**b**) The detail of quantum vibration on the quantum platform of I-V curve on the pillars sample II, in which the twin peaks in the PL spectrum indicate the twin states in the localized states; (**c**) The change of the quantum vibration in magnetic field with 0.33T (red curve) on the sample II.

It is amazed that the quantum vibration on the quantum platform of I-V curve is discovered on the pillars structure doped with oxygen, as shown in Fig. 9(a), in which the quantum effect only occurs on the sample II owing to the localized states related to the red curve. And the quantum twin-state occurs in the localized states in which the vibration of photo-generated current forms under irradiation of laser at 633 nm, as shown in Fig. 9(b). Here, the energy difference ∆E in the quantum twin-state is about 10 meV as exhibited in the twin peaks of PL spectrum in the inset of Fig. 9(b). The resonance between the twin states in light field has the characteristic of the quantum vibration confined in the localized states. It should be noted that the amplitude change of the quantum vibration occurs on the sample II in the magnetic field with 0.33T as shown in Fig. 9(c), which is related to the electron spin in the localized states. The quantum twin-state in the localized states should has an application in quantum entangle and quantum code.

**Figure 10:**
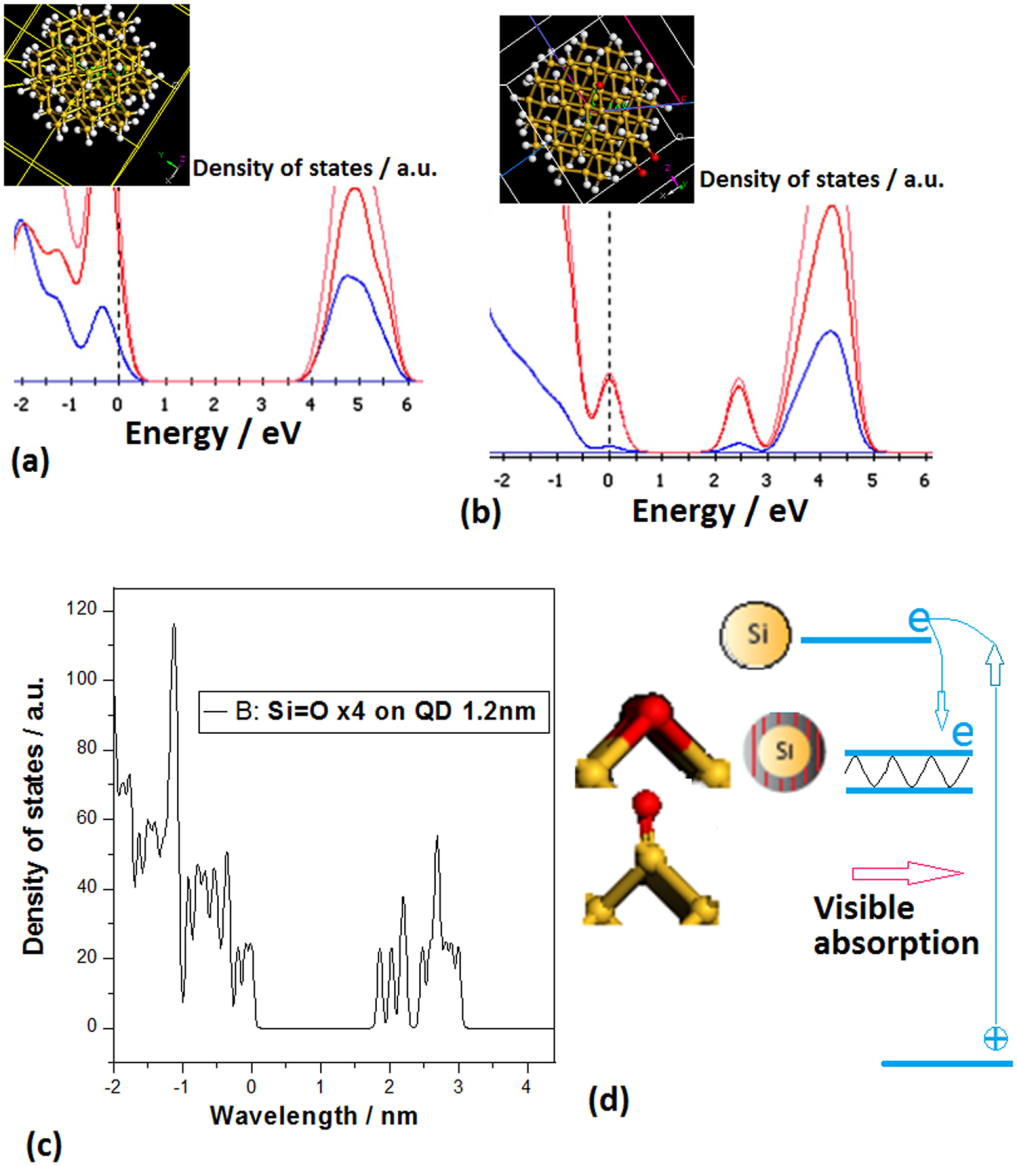
(**a**) The model structure of Si quantum dot (the inset:Si atom(yellow ball) and hydrogen atom(white ball)) and the DOS distribution in the first principle calculation; (**b**) The model structure of quantum dot doped with oxygen (the inset:Si atom(yellow ball), oxygen atom(red ball) and hydrogen atom(white ball)) and the DOS distribution in the first principle calculation, in which the localized states occur in the band gap; (**c**) The DOS distribution involving the localized states in the band gap in the first principle calculation related to Si=O bonds on Si QDs surface; (**d**) The forming model of the localized states on the pillars sample II prepared in oxygen.

In the simulating calculation, the structure models of the Si quantum dot and the impuritied quantum dot have been built in order to simulate the experiment process, as shown in the inset of Fig. 10(a) and (b). An abinitio non-relativistic quantum mechanical analysis is used to investigate the electronic behavior. The DFT calculation is carried out by using the local density approximation (LDA) and the generalized gradient approximation (GGA) for the self-consistent total energy calculation. It is interested to make a comparison between Fig. 10(a) and (b), in which the localized states originated from the quantum dot doped with oxygen appear in the band gap of the density of states (DOS) just in Fig. 10(b). Figure 10(c) shows the DOS distribution on Si quantum dot with four Si=O bonds on surface in the simulating calculation, where it should be noted that the fine split structure occurs in the localized states owing to Si=O bonds. Therefore, the physical model for explaining the localized states effect on the quantum dot doped with oxygen is built in Fig. 10(d), in which the spin split effect can be amplified in the fine split structure of localized states under the magnetic field.

In conclusion, the multiple impuritied nanolayers on the pillars structure have been fabricated for Si solar cells to pick up photons in ultraviolet and infrared region of solar spectra. The localized states with longer lifetime originated from impurities on nanosilicon effectively avoid Auger recombination in the photovoltaic system. The calculation results indicate that the localized states occur in various regions on the pillars structure with the multiple impuritied nanolayers, which is demonstrated in the PL spectra measurement. The quantum effect of photo-generated carriers is observed in I-V curve measurement on the photovoltaic sample prepared in oxygen. The quantum vibration on the I-V quantum platform is discovered on the pillars structure doped with oxygen, and the quantum twin-state occurs in the localized states under irradiation of laser at 633 nm, in which the amplitude change of the quantum vibration appears in magnetic field related to the electron spin in the localized states. This kind of quantum twin-state in the localized states should have an application in quantum code and quantum entanglement in the photo-communication.

## Methods

### Fabrication of the pillars structure by using nanosecond pulsed laser

A silicon wafer of P-type (100) oriented substrate with 1–10 Ωcm is taken on the sample stage in the fabrication system with nanosecond pulsed laser etching (ns-PLE) devices. A third harmonic of pulsed Nd:YAG laser at 355 nm with nanosecond pulsed width is used to etch Si surface by controlling with points and lines array scanning in vacuum or in various environment. For making a comparison between the samples prepared in different environment, we fabricate the pillars sample I prepared in vacuum to build the pure nanosilicon and the pillars sample II prepared in oxygen of 80 Pa to build the impurities structure on nanosilicon surface. After the etching process, in the amorphous layer on the pillars surface, the Si QDs can grow up through crystallizing in the irradiation of electron beam or the laser annealing and the furnace annealing process, in which their size range is narrower due to layer thickness confinement. The doping process with oxygen can be carried out through the gas tube in the chamber of the PLE combination fabrication system.

### Preparation of multiple nanolayers impuritied on the pillars structure

On the pillars surface, the nanolayers can be prepared in the combination fabrication system with PLE and pulsed laser deposition (PLD) devices. A third harmonic of pulsed Nd:YAG laser at 355 nm is used to deposit nanolayers on the pillars in PLD process in various environment. The smaller QDs in the ultraviolet nanolayer (layer thickness: 2–3 nm) and the bigger QDs in the visible nanolayer (layer thickness: 3–5 nm) can be fabricated by using PLD method and controlling the annealing time. The defects in the infrared nanolayer (layer thickness: 8–10 nm) can be prepared by the faster annealing after PLD process. The doping process on the nanosilicon or on the defects can be carried out in the PLD method with oxygen environment for producing the localized states.

### Photoluminescence (PL) measurement

PL spectra on the pillars samples are measured under the 266 nm or 488 nm excitation at room temperature (300 K) and lower temperature (10 K ~ 200 K) in sample chamber of 1 Pa. In the PL measurement system, The ultraviolet laser beam at 266 nm is used to measure the ultraviolet spectrum, and the light beam at 488 nm in Ar ion laser is focused on the pillars samples for excitation to detect the visible spectrum and the infrared spectrum for identifying the electronic states on the impuritied QDs or on the defects doped with oxygen.

### Photovoltaic measurement

The construction of photovoltaic system is shown in Fig. 2(a), in which the PIN structure involves the bottom buffer silicon layer on p-type silicon substrate, the multiple nanolayers N-type doped with oxygen in which the nanosilicon such as the impuritied QDs and the defects are confined to form the inter-layer, and the top pillars surface. The positive pole is connected on the gold film under the P-type Si layer and the negative pole is connected with the ITO film on the N-type Si top layer. The I-V character of the PIN junction is measured with voltage from −10 V to 15 V, in which the threshold effect and the quantum tunneling effect are obviously observed. The negative resistance phenomenon and the resonant vibration between the twin states in light field are measured, which have the characteristic of the quantum vibration confined in the localized states. The amplitude change of the quantum vibration with ∆E~10 meV is observed on the pillars sample II prepared in oxygen in magnetic field with 0.33 T, which is related to the electron spin in the localized states.

## References

[1] Zhao J, Wang A, Green MA, Ferrazza F (1998). Novel 19.8% efficient ‘honeycomb’ textured multicrystalline and 24.4% monocrystalline silicon solar cells. Appl. Phys. Lett..

[2] Shockley W, Queisser HJ (1961). Detailed balance limit of efficiency of p–n junction solar cells. J.Appl. Phys..

[3] Polman A, Atwater HA (2012). Photonic design principles for ultra-high efficiency photovoltaics. Nature Mater..

[4] Sanjay K (2010). Husain, Excellent antireflection properties of vertical silicon nanowire arrays, Solar Energy Material Solar Cells.

[5] Kumar, D., Srivastava, S. K., Singh, P. K., Husain, M., Kumar, V. Fabrication of silicon nanowire arrays based solar cell with improved performance,Solar Energy Material Solar Cells, Vol. 95, 215–218 (2011).

[6] Gur I, Fromer NA, Geier ML, Alivisatos AP (2005). Air-stable all-inorganic nanocrystal solar cells processed from solution. Science.

[7] O’Regan B, Gra¨tzel M (1991). A low-cost, high-efficiency solar cell based on dye-sensitized colloidal TiO2 films. Nature.

[8] Law M, Greene LE, Johnson JC, Saykally R, Yang P (2005). Nanowire dye-sensitized solar cells. Nat. Mater..

[9] Tang J (2011). Colloidal-quantum-dot photovoltaics using atomic-ligand passivation. Nat. Mater..

[10] Wallentin J (2013). InP nanowire array solar cells achieving 13.8% efficiency by exceeding the ray optics limit. Science.

[11] Tang J, Huo Z, Brittman S, Gao H, Yang P (2011). Solution-processed core–shell nanowires for efficient photovoltaic cells. Nat. Nanotech..

[12] Zhu J, Hsu C-M, Yu Z, Fan S, Cui Y (2010). Nanodome solar cells with efficient light management and self-cleaning. Nano Lett..

[13] Kelzenberg MD (2010). Enhanced absorption and carrier collection in Si wire arrays for photovoltaic applications. Nat. Mater..

[14] Garnett EC, Brongersma ML, Cui Y, McGehee MD (2011). Nanowire solar cells. Annu. Rev. Mater. Res..

[15] Yoon J (2008). Ultrathin silicon solar microcells for semitransparent, mechanically flexible and microconcentrator module designs. Nat. Mater..

[16] Fan Z (2009). Three-dimensional nanopillar-array photovoltaics on low-cost and flexible substrates. Nat. Mater..

[17] Tian B (2007). Coaxial silicon nanowires as solar cells and nanoelectronic power sources. Nature.

[18] Goodrich AC, Powell DM, James TL, Woodhouse M, Buonassisi T (2013). Assessing the drivers of regional trends in solar photovoltaic manufacturing. Energy Environ. Sci..

[19] U.S. Department of Energy. SunShot Vision Study. 69–96 (2012).

[20] Mavrokefalos A, Han SE, Yerci S, Branham MS, Chen G (2012). Efficient light trapping in inverted nanopyramid thin crystalline silicon membranes for solar cell applications. Nano Lett..

[21] Powell DM (2012). Crystalline silicon photovoltaics: a cost analysis framework for determining technology pathways to reach baseload electricity costs. Energy Environ. Sci..

[22] Kim DR, Lee CH, Rao PM, Cho IS, Zheng X (2011). Hybrid Si microwire and planar solar cells: passivation and characterization. Nano Lett..

[23] Garnett E, Yang P (2010). Light trapping in silicon nanowire solar cells. Nano Lett..

[24] Kempa TJ (2012). Coaxial multishell nanowires with high-quality electronic interfaces and tunable optical cavities for ultrathin photovoltaics. Proc. Natl Acad. Sci. USA.

[25] Zhu J (2009). Optical absorption enhancement in amorphous silicon nanowire and nanocone arrays. Nano. Lett..

[26] Wang KX, Yu Z, Liu V, Cui Y, Fan S (2012). Absorption enhancement in ultrathin crystalline silicon solar cells with antireflection and light-trapping nanocone gratings. Nano Lett..

[27] Jeong S (2012). Hybrid Si nanocone-polymer solar cells. Nano. Lett..

[28] Leung S-F (2012). Efficient photon capturing with ordered three-dimensional nanowell arrays. Nano. Lett..

[29] Han SE, Chen G (2010). Optical absorption enhancement in silicon nanohole arrays for solar photovoltaics. Nano. Lett..

[30] Peng K-Q, Wang X, Li L, Wu X-L, Lee S-T (2010). High-performance silicon nanohole solar cells. J. Am. Chem. Soc..

[31] Oh J, Yuan H-C, Branz HM (2012). An 18.2%-efficient black-silicon solar cell achieved through control of carrier recombination in nanostructures. Nat. Nanotech..

[32] Lu Y, Lal A (2010). High-efficiency ordered silicon nano-conical-frustum array solar cells by self-powered parallel electron lithography. Nano. Lett..

[33] Huang W-Q (2016). Lasing with Pumping Levels of Si Nanocrystals on Silicon Wafer. Nanoscale Research Letters.

[34] Huang, W.-Q., Huang, Z.-M. & Liu, S.-R. Electronic states of defect with impurity and infrared emission on black Silicon prepared by an ns-laser. *Optics Letters* **42**(2), 358 (2017). 10.1364/OL.42.000358 28081112

[35] Luryi S (1985). Frequency limit of double-barrier resonant-tunneling oscillators. Appl.Phys.Lett..

